# Impact of Sodium–Glucose Cotransporter 2 Inhibitors on Acute Kidney Injury Post Off-Pump Coronary Artery Bypass Grafting: A Retrospective Cohort Study and Meta-Analysis

**DOI:** 10.31083/RCM39400

**Published:** 2026-01-21

**Authors:** Xiaozheng Zhou, Yilin Pan, Kun Hua, Xiubin Yang

**Affiliations:** ^1^Department of Cardiovascular Surgery, Beijing Anzhen Hospital, Capital Medical University, Beijing Institute of Heart, Lung and Vessel Disease, 100029 Beijing, China

**Keywords:** AKI, SGLT2is, OPCABG, in-hospital death, inflammatory

## Abstract

**Background:**

Sodium–glucose cotransporter 2 (SGLT2) inhibitors, a novel class of oral antihyperglycemic medications prescribed for type 2 diabetes mellitus, play a beneficial role in slowing the progression of heart failure. However, debate persists regarding the potential link of these inhibitors to acute kidney injury (AKI) in specific clinical conditions.

**Methods:**

This study was a retrospective analysis of consecutive patients receiving off-pump coronary artery bypass grafting (OPCABG) at our institution between January 2018 and July 2023. A group of patients who had been administered SGLT2 inhibitors was systematically compared with non-users in a 1:3 ratio using propensity score matching. The principal endpoint was postoperative AKI after OPCABG. In addition, we performed a comprehensive meta-analysis of the associations between SGLT2 inhibitor therapy and AKI risk. The analytical approach combined institutional data with aggregated findings from existing literature.

**Results:**

The analysis encompassed 403 patients who administered SGLT2 inhibitors and 1209 non-users. AKI developed in 54 cases (13.4%) post-OPCABG among individuals who received SGLT2 inhibitors, compared to 373 cases (30.9%) in the control cohort. Statistical analysis demonstrated significantly reduced AKI prevalence in the SGLT2 inhibitor cohort compared to non-users (*p* < 0.001). The meta-analysis results confirmed a protective association between SGLT2 inhibitor therapy and AKI risk reduction (odds ratio (OR) = 0.525, 95% confidence interval (CI) 0.437–0.631; *p* < 0.001).

**Conclusion:**

In this study, SGLT2 inhibitor administration was associated with a decreased incidence of postoperative AKI in OPCABG patients.

**Clinical Trial Registration:**

NCT05888168, https://clinicaltrials.gov/study/NCT05888168?cond=NCT05888168&rank=1.

## 1. Introduction

Coronary artery disease (CAD) is commonly associated with multi-organ dysfunction and is recognized as a major contributor to mortality in hospitalized critically ill patients [1, 2, 3]. For patients presenting with left main coronary artery obstructions or three-vessel disease, the primary therapeutic approach for CAD is coronary artery bypass grafting [4]. A major postoperative concern following off-pump coronary artery bypass surgery (OPCABG) is acute kidney injury (AKI), which is associated with increased a common mortality and length of hospital stay [3, 5, 6].

Sodium-glucose cotransporter 2 inhibitors (SGLT2is), including dapagliflozin, represent a novel category of oral medications for diabetes that demonstrate substantial cardiovascular benefits by lowering the incidence of heart failure and mortality among diabetic patients and those with reduced ejection fraction [7, 8, 9]. Emerging evidence suggests these pharmacological agents might effectively mitigate acute kidney injury associated with percutaneous coronary interventions (PCI-AKI) [10]. Multiple systematic reviews have examined both therapeutic outcomes and adverse effects of SGLT2is in patients with acute kidney injury [9, 11, 12]. However, the association between SGLT2 inhibitors and reduced AKI following OPCABG requires further clarification. This research aims to evaluate the potential of SGLT2s in diminishing the risk of OPCABG-AKI and their role in decreasing postoperative mortality.

## 2. Methods

### 2.1 Study Population 

This retrospective analysis examined consecutive patients undergoing OPCABG at our institution from January 2018 to July 2023. The trial was registered on ClinicalTrials.gov (NCT05888168, https://clinicaltrials.gov/study/NCT05888168?cond=NCT05888168&rank=1). Ethical clearance was obtained from the ethics committee at Beijing Anzhen Hospital (2023065X), following the principles of the Declaration of Helsinki. The research protocol incorporated informed consent provisions with authorized waivers and adhered to the STROCSS reporting standards [13]. AKI and AKIN (three stages of AKI), were the primary end points of the study. The diagnosis of AKI was determined by a serum creatinine level ≥0.3 mg/dL (26.2 µmol/L) or 1.5–2-fold greater than the baseline level in the first 48 hours after OPCABG [3]. Baseline creatinine was defined as the most recent preoperative serum creatinine measurement obtained within 24 hours prior to surgery. Patients without a preoperative value within this window were excluded per study protocol (see exclusion criteria). The following criteria were used to exclude candidates: Age <18 or >80 years; preoperative dialysis dependency or end-stage renal disease estimated glomerular filtration rate (eGFR) <15 mL/min/1.73 m^2^); severe comorbidities: decompensated cirrhosis (Child-Pugh C) or acute liver failure, metastatic malignancy, NYHA Class IV heart failure or cardiogenic shock, or severe chronic obstructive pulmonary disease (COPD) (forced expiratory volume in 1 second, FEV1 <50% predicted); history of major non-cardiac surgery within 3 months prior to OPCABG (e.g., nephrectomy, bowel resection, neurosurgery); or an active systemic infection requiring intravenous antibiotics at the time of surgery.

SGLT2i treatment protocol: patients in the SGLT2i group met the following criteria: medication regimen: Dapagliflozin 10 mg once daily (other SGLT2is were excluded for homogeneity); treatment duration: Minimum 3 months of uninterrupted therapy preoperatively; Preoperative Discontinuation: Stopped 72 hours before surgery.

### 2.2 Data Collection

The study incorporated demographic and clinical variables encompassing patient age, gender, body composition metrics (BMI), pre-existing medical conditions, and preoperative laboratory values including serum creatinine levels and eGFR. Additional perioperative parameters that were analysed included postoperative atrial fibrillation occurring within 72 hours, duration of intensive care unit (ICU) stay, total treatment expenditures, and in-hospital mortality. The primary outcome was the occurrence of AKI defined as either a serum creatinine elevation ≥0.3 mg/dL (26.2 µmol/L) or 1.5–2 time increase from baseline within 48 hours following OPCABG. Secondary endpoints included in-hospital mortality from any cause, requirement for continuous renal replacement therapy (CRRT), postoperative ICU length of stay, and total hospital stay.

### 2.3 Statistical Analysis

Continuous variables were expressed as data means, while categorical variables were shown in percentage form. Quantile-quantile (QQ) plots were utilized to evaluate the normality of distributions. To address baseline discrepancies, a 1:3 propensity score matching (PSM) analysis was conducted between SGLT2i and non-SGLT2i groups using R software (http://www.R-project.org), incorporating multiple covariates for balanced comparisons. The variables incorporated into PSM calculations included hypertension status, presence of hyperlipidemia, uric acid levels, prior PCI history, ejection fraction, white blood cell counts, and pre-operative medication regimens. This matching methodology aimed to minimize potential confounding factors between treatment cohorts through systematic data pairing.

To address baseline disparities between the SGLT2i and non-SGLT2i cohorts, a propensity score matching approach was employed. The propensity score represented the probability conditioned on receiving SGLT2i therapy when analysed as a dichotomous outcome variable, aiming to mitigate inherent selection bias and address confounding variables. The matching model incorporated these covariates: history of hypertension, hyperlipidemia status, elevated uric acid levels, prior percutaneous coronary intervention (PCI), ejection fraction (EF), white blood cell (WBC), and preoperative drug regimens. Using a nearest-neighbor methodology, patients were sequentially paired based on descending propensity scores. A greedy matching algorithm with a 0.05 standard deviation caliper restriction was implemented for 1:3 ratio matching between groups, excluding replacement. Unmatched participants meeting no suitable counterparts were excluded from subsequent analysis.

To evaluate the relationship between SGLT2 inhibitor usage and AKI incidence within this cohort, multiple-variable logistic regression models were conducted. The predetermined threshold for statistical significance was set at *p* < 0.05.

### 2.4 Meta-Analysis

A comprehensive literature search was conducted across PubMed, EMBASE, and the Cochrane Library (detailed search terms available in **Supplementary Table 1**). Trial registries listed on ClinicalTrials.gov were further scrutinized to identify reports of severe acute kidney injury/renal failure in SGLT2 inhibitor studies. All search parameters, eligibility criteria, and data extraction protocols had been predefined in the study protocol and maintained without modification throughout the research process. To ensure thoroughness, manual screening of reference sections from relevant publications and review articles about SGLT2 inhibitors was performed (illustrated in **Supplementary Fig. 1**). This investigation followed the Systematic Reviews and Meta-Analyses (PRISMA) guidelines for systematic reviews and meta-analyses. Two researchers (PYL and XZZ) independently extracted study data using standardized forms across multiple databases. Any disagreements in data interpretation were resolved through collaborative discussions among all study authors. The principal outcome measure focused on the occurrence of acute kidney injury.

The data were sourced from peer-reviewed publications and data available on ClinicalTrials.gov. Software used for meta-analysis: we used Stata MP 18.0 (StataCorp, College Station, TX, USA) for all statistical analyses. Effect size selection: We selected the odds ratio (OR) with a random-effects model using the Mantel-Haenszel method as the primary effect measure for dichotomous outcomes (AKI incidence), as this is the most conservative and widely recommended approach for clinical study meta-analyses where some heterogeneity is expected. The relevant information included in the study can be found in **Supplementary Table 2**. The search strategy was formulated by integrating the keywords “AKI” and “SGLT2i”. Intergroup differences were evaluated using the Z test, with statistical significance defined as a two-tailed *p*-value threshold of <0.05.

## 3. Results

### 3.1 Populations and Clinical Characteristics

**Fig. 1.**
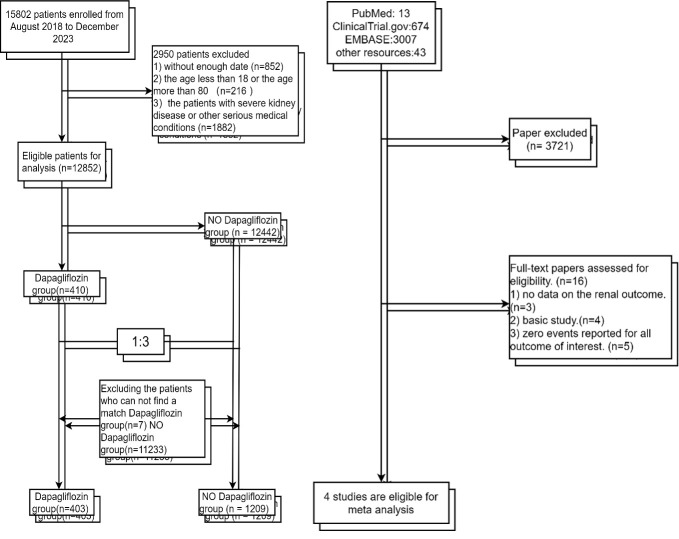
**Study design**. The research flowchart consists of two parts. One part provides the data from our research center, and the other part is a meta-analysis of previous studies.

During the investigation period, the study initially enrolled 15,802 OPCABG recipients. Application of exclusion criteria led to the removal of 2950 participants from the analysis. Within the final study cohort, preoperative dapagliflozin administration for diabetes or heart failure management was documented in 410 cases. Post-PSM implementation, seven individuals from the SGLT2i cohort were eliminated due to unsuccessful matching with non-SGLT2i counterparts. The matching process yielded 403 dapagliflozin-treated subjects and 1209 controls in the non-dapagliflozin group through 1:3 PSM, as shown in Fig. 1.

The characteristics of the patients are presented in Table 1. The majority of variables in the baseline data did not demonstrate any significant differences between the two groups. Before PMS, the dapagliflozin group had significantly higher rates of hypertension (66.6% vs. 58.2%, *p* = 0.001) and hyperlipidemia (70.2% vs. 51.1%, *p* < 0.001), and lower EF (56.56% vs. 59.49%, *p* < 0.001) than the non-dapagliflozin group. After 1:3 PMS, all baseline characteristics including hypertension (66.3% vs. 65.1%, *p* = 0.672) and hyperlipidemia (70.0% vs. 68.5%, *p* = 0.576) were balanced (all *p* > 0.05), as shown in Table 1. The distribution of covariates was evaluated via standard mean difference and chi-square tests, which demonstrated that all covariates were balanced by PSM, as illustrated in Table 1. Most of the patients were treated with diabetes and heart failure, including heart failure with preserved ejection fraction and heart failure with reduced ejection fraction.

**Table 1.** **Baseline characteristics**.

Variable		Before propensity score matching			After propensity score matching		
		Dapagliflozin (n = 410)	No Dapagliflozin (n = 12,442)	*p*	Dapagliflozin (n = 403)	No Dapagliflozin (n = 1209)	*p*
Age, years		61.86 ± 8.51	62.19 ± 8.71	0.456	61.81 ± 8.52	62.05 ± 8.69	0.638
BMI, kg/m^2^		25.61 ± 3.35	25.83 ± 4.36	0.297	25.57 ± 3.36	25.78 ± 3.27	0.272
Male sex, n (%)		322 (78.5%)	9368 (75.3%)	0.134	316 (78.4%)	942 (77.9%)	0.835
Hypertension, n (%)		273 (66.6%)	7247 (58.2%)	0.001	267 (66.3%)	787 (65.1%)	0.672
Diabetes, n (%)		137 (33.4%)	4338 (34.9%)	0.544	134 (33.3%)	357 (29.5%)	0.160
Hyperlipidemia, n (%)		288 (70.2%)	6355 (51.1%)	<0.001	282 (70%)	828 (68.5%)	0.576
COPD, n (%)		1 (0.2%)	49 (0.4%)	0.939	1 (0.2%)	6 (0.5%)	0.827
Smoke, n (%)		98 (23.9%)	2873 (23.1%)	0.701	97 (24.1%)	243 (20.1%)	0.091
Alcohol, n (%)		93 (22.7%)	2618 (21%)	0.423	91 (22.6%)	230 (19%)	0.122
High uric acid, n (%)		107 (26.1%)	4295 (34.5%)	<0.001	106 (26.3%)	310 (25.6%)	0.793
Hepatic insufficiency, n (%)		4 (1%)	94 (0.8%)	0.829	4 (1%)	12 (1%)	1.000
Cerebral infarction, n (%)		45 (11%)	1381 (11.1%)	0.937	44 (10.9%)	136 (11.2%)	0.855
PCI, n (%)		64 (15.6%)	1167 (9.4%)	<0.001	62 (15.4%)	202 (16.7%)	0.534
The number of Narrow coronaries <3, n (%)		157 (38.3%)	4651 (37.4%)	0.141	153 (38%)	513 (42.4%)	0.115
Pulmonary infection		22 (5.4%)	481 (3.9%)	0.123	21 (5.2%)	54 (4.5%)	0.539
MBP (mmHg)		94.8 ± 23.83	93.28 ± 12.05	0.201	94.75 ± 23.9	93.53 ± 12	0.201
Renal artery stenosis n (%)		1 (0.2%)	74 (0.6%)	0.556	1 (0.2%)	8 (0.7%)	0.563
Preoperative medications							
	Aspirin	126 (30.7%)	3569 (28.7%)	0.368	124 (30.8%)	330 (27.3%)	0.179
	Beta-blockers, n (%)	168 (41%)	3355 (27%)	<0.001	161 (40%)	481 (39.8%)	0.953
	ACE inhibitors/ARB, n (%)	178 (43.4%)	2178 (17.5%)	<0.001	171 (42.4%)	480 (39.7%)	0.333
	Diuretics, n (%)	186 (45.4%)	3079 (24.7%)	<0.001	179 (44.4%)	532 (44%)	0.885
	CCB, n (%)	108 (26.3%)	1955 (15.7%)	<0.001	105 (26.1%)	314 (26%)	0.974
	Rate, n	80.78 ± 15.74	80.01 ± 16.26	0.343	80.66 ± 15.72	79.75 ± 16.1	0.325
	LVDD, mm	33.36 ± 6.57	32.71 ± 6.08	0.032	33.35 ± 6.50	33.41 ± 6.65	0.869
	EF, %	56.56 ± 9.66	59.49 ± 8.62	<0.001	56.66 ± 9.61	56.99 ± 9.78	0.559
	TG, mmol/L	1.73 ± 1.08	1.67 ± 1.04	0.265	1.73 ± 1.08	1.71 ± 1.03	0.679
	WBC, 10^9^/L	9.04 ± 3.76	8.12 ± 3.21	<0.001	8.98 ± 3.71	8.82 ± 3.77	0.461
	Hb, g/L	130.79 ± 22.11	130.77 ± 20.94	0.984	130.84 ± 22.17	130.4 ± 21.12	0.720
	eGFR, mL/min	89.68 ± 16.73	90.42 ± 16.03	0.353	89.69 ± 16.85	90.51 ± 15.93	0.374
	PLT, 10^9^/L	211.58 ± 65.63	215.13 ± 65.46	0.280	212.1 ± 65.48	214.57 ± 66.82	0.518
	Lactic acid, mmol/L	1.6 ± 0.69	1.65 ± 0.82	0.208	1.6 ± 0.69	1.68 ± 0.83	0.078
	Potassium, mmol/L	4.05 ± 0.40	4.07 ± 0.38	0.282	4.04 ± 0.40	4.06 ± 0.37	0.353
	Calcium, mmol/L	2.3 ± 0.10	2.3 ± 0.11	0.742	2.3 ± 0.10	2.31 ± 0.11	0.064
	Magnesium	0.9 ± 0.08	0.89 ± 0.08	0.418	0.9 ± 0.08	0.9 ± 0.08	0.706
	Creatine, µmol/L	76.68 ± 20.18	75.98 ± 37.53	0.708	76.47 ± 20.12	75.61 ± 37.23	0.658
	Uric acid, µmol/L	314.74 ± 87.34	323.25 ± 95.8	0.054	314.15 ± 87.69	312.2 ± 94.52	0.715
	Glucose, mmol/L	6.77 ± 3.18	6.6 ± 2.63	0.294	6.74 ± 3.17	6.82 ± 2.91	0.658

BMI, body mass index; COPD, chronic obstructive pulmonary disease; PCI, percutaneous coronary intervention; ACE, angiotensin converting enzyme; ARB, angiotensin receptor blocker; CCB, calcium channel blocker; LVDD, left ventricular end diastolic dimension; EF, ejection fraction; TG, triglyceride; WBC, white blood cell; Hb, hemoglobin; PLT, platelet; eGFR, estimated glomerular filtration rate; MBP, mean blood pressure.

### 3.2 Relationship Between Dapagliflozin and Outcomes After OPCABG

Univariate logistic regression analysis revealed that patients in the dapagliflozin group had less AKI and a lower in-hospital mortality, and patients were less likely to use intra-aortic balloon pump (IABP) and CRRT, as shown in in **Supplementary Table 3**. After PSM, the dapagliflozin treatment group was associated with a lower risk of AKI after OPCABG than the no dapagliflozin treatment group [54 (13.4%) vs. 373 (30.9%); *p* < 0.001]; the same was found for in-hospital mortality [5 (1.2%) vs. 38 (3.1%); *p* < 0.048] (Table 2).

**Table 2.** **Clinical outcomes and postoperative complications after PMS**.

Variable		Dapagliflozin (n = 403)	No Dapagliflozin (n = 1209)	The all	*p*	OR (95% CI)	*p*-value
Death		5 (1.2%)	38 (3.1%)	43 (2.7%)	0.048	0.387 (0.151∼0.990)	0.038
AKI		54 (13.4%)	373 (30.9%)	427 (26.5%)	<0.001	0.347 (0.254∼0.474)	<0.001
KDIGO					<0.001		<0.001
	0	349 (86.6%)	836 (69.1%)	1185 (73.5%)		2.884 (2.112∼3.938)	
	1	30 (7.4%)	208 (17.2%)	238 (14.8%)		0.387 (0.259∼0.578)	
	2	15 (3.7%)	96 (7.9%)	111 (6.9%)		0.403 (0.231∼0.703)	
	3	9 (2.2%)	69 (5.7%)	78 (4.8%)		0.248 (0.123∼0.501)	
POAF		113 (28%)	410 (33.9%)	523 (32.4%)	<0.001	0.759 (0.593∼0.973)	0.051
CRRT		4 (1%)	43 (3.6%)	47 (2.9%)	0.008	0.272 (0.097∼0.762)	0.010
IABP		11 (2.7%)	72 (6%)	83 (5.1%)	0.011	0.443 (0.233∼0.844)	0.009

AKI, acute kidney injury; KDIGO, Kidney Disease Improving Global Outcomes; POAF, postoperative atrial fibrillation; CRRT, continuous renal replacement therapy; IABP, intraaortic balloon pump; ECMO, extra corporeal membrane oxygenation. *p*: The results of the univariate analysis; *p*-value: The results of the logistic regression analysis.

### 3.3 Subgroup Analysis

**Fig. 2.**
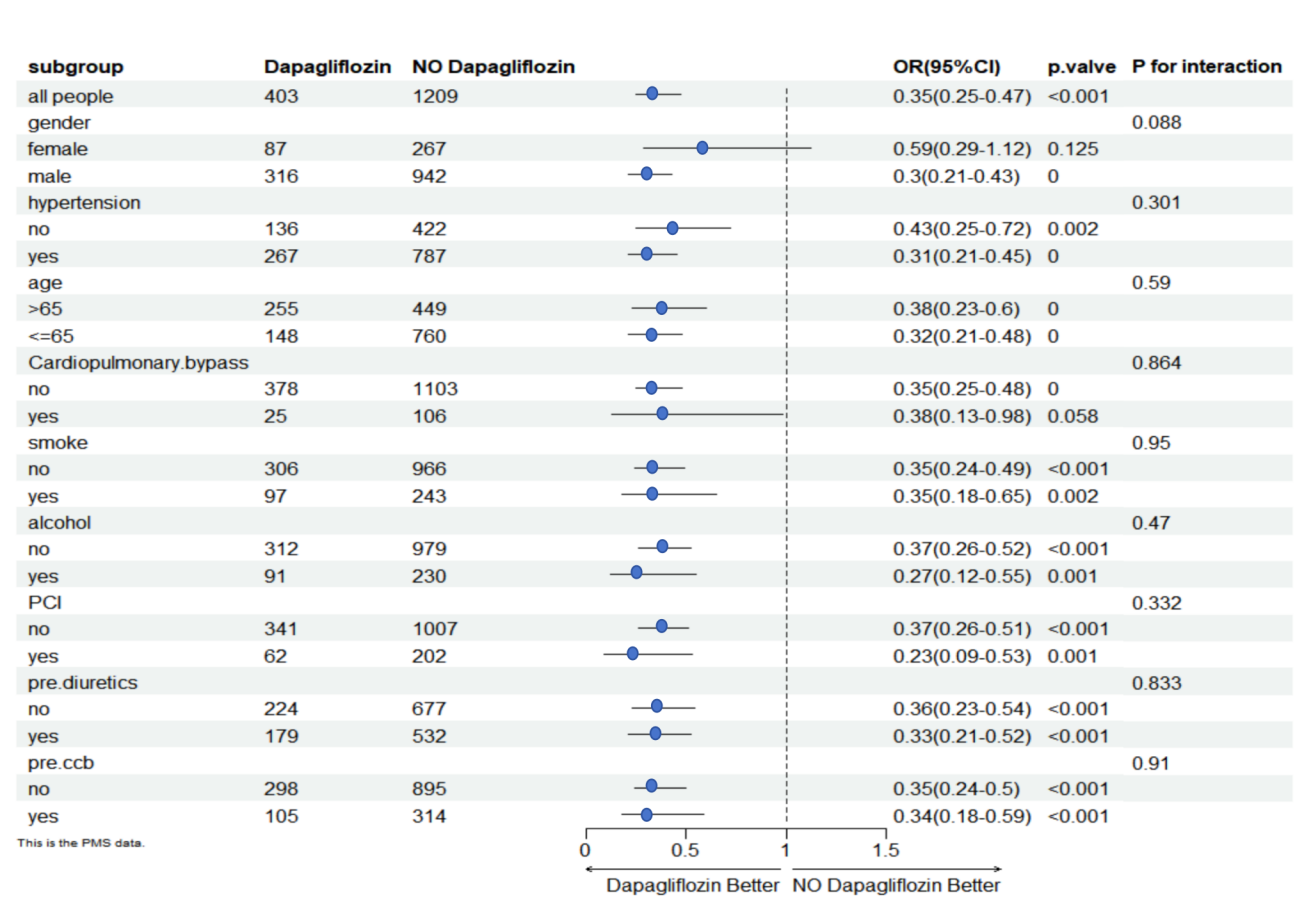
**Subgroup analysis**. OR, odds ratio; PCI, percutaneous coronary intervention; ccb, calcium channel blocker.

The risk of AKI in CAD patients was evaluated in different subgroups by hyperlipidaemia, high uric acid, PCI, EF, WBC, hypertension, and preoperative medications (Fig. 2). Subgroup analysis revealed the efficacy of SGLT2is on AKI-OPCABG compared to not receiving SGLT2is. The effectiveness of dapagliflozin in treating AKI-OPCABG was not significantly different between subgroups (*p* for interaction >0.05).

### 3.4 Meta-Analysis

**Fig. 3.**
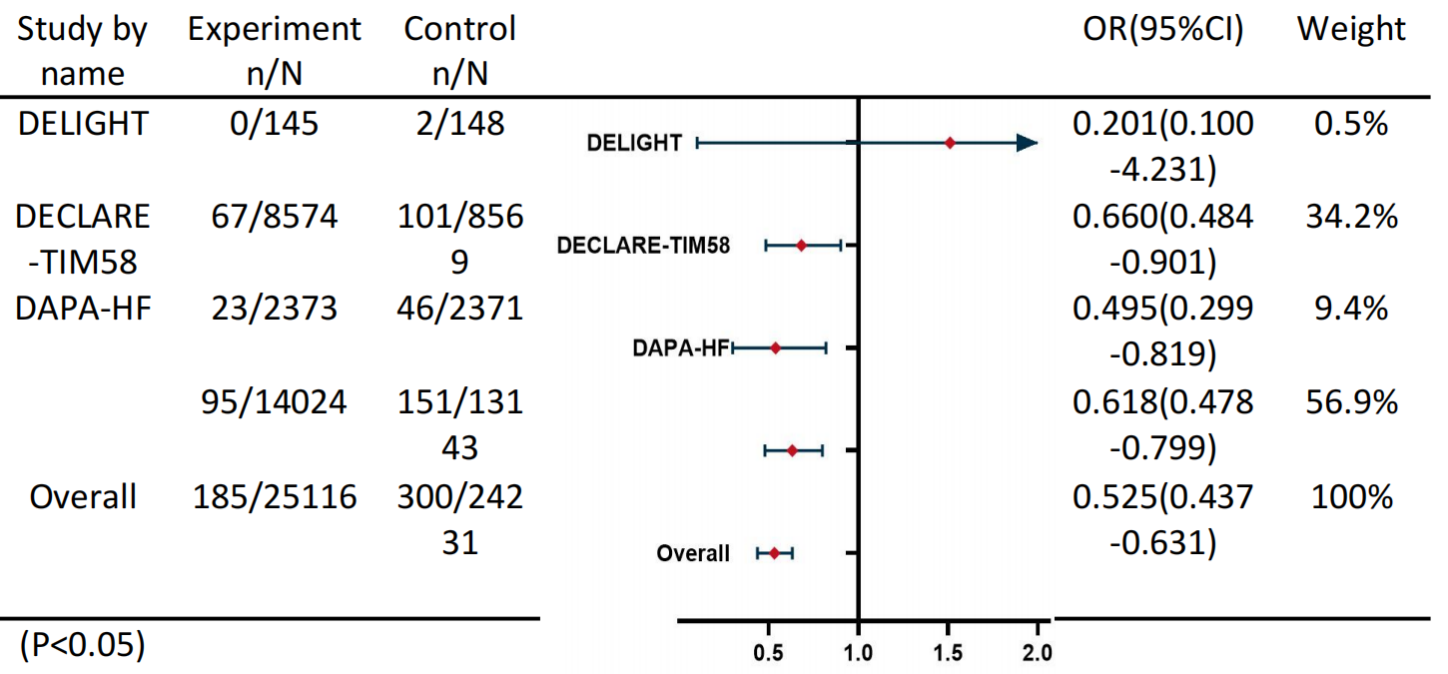
**Meta-Analysis**. N: The total sample size included in the study; n: The number of patients with AKI.

The meta-analysis initially identified 3737 studies through a comprehensive search of PubMed, ClinicalTrial.gov, EMBASE, and other relevant resources. Following the removal of duplicates, the titles, abstracts, and full-text articles were subjected to a screening process to identify all potentially eligible studies for inclusion. The inclusion criteria were met by a total of four studies, which were then subjected to a systematic analysis in the context of this meta-analysis (Fig. 3). The results of the meta-analysis indicated that the SGLT2i group was associated with a lower incidence of AKI than the non-SGLT2i group was (OR, 0.525 [95% CI, 0.437–0.631]; I^2^ = 0.0%).

### 3.5 Postoperative Requirement of CRRT

**Fig. 4.**
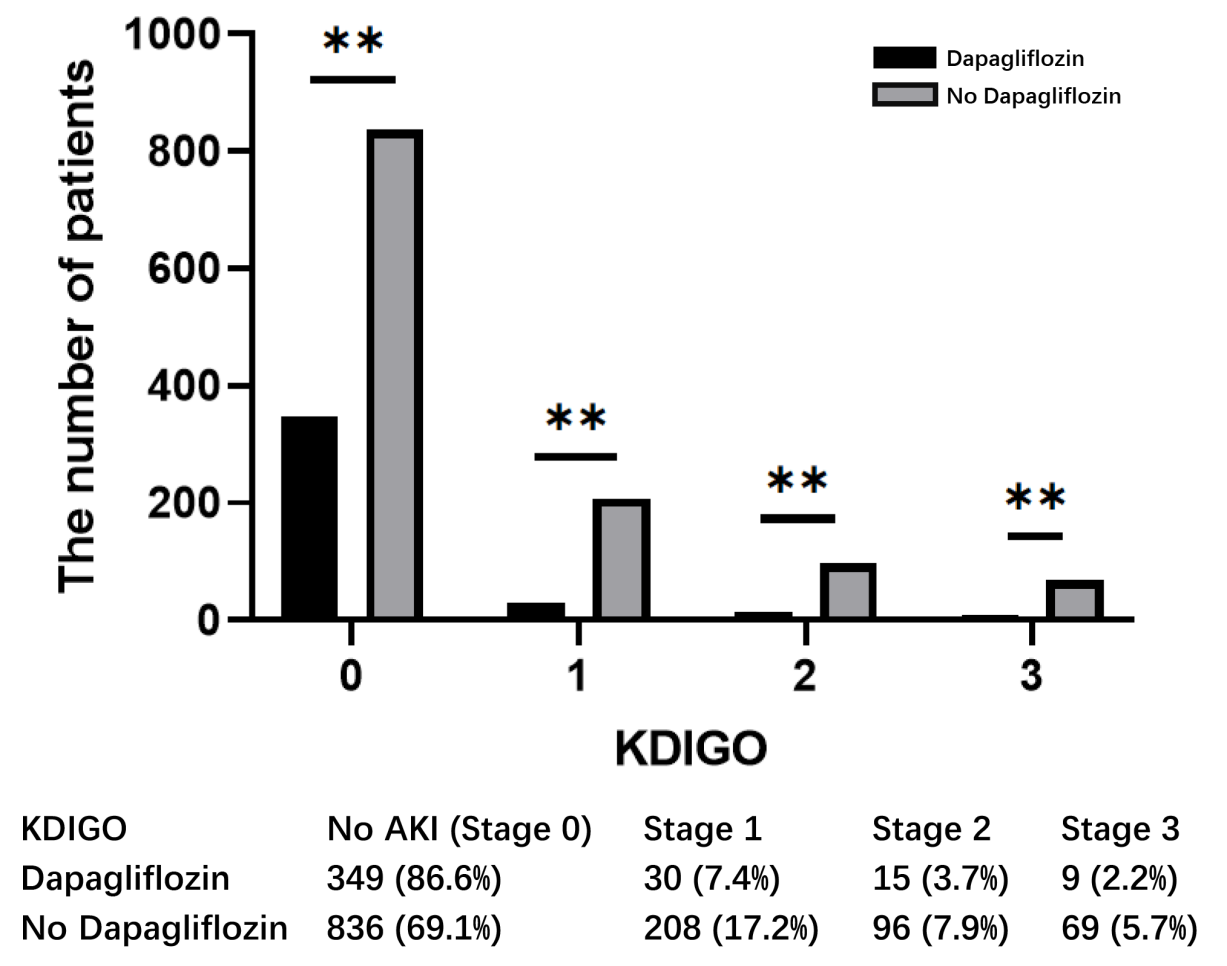
**KDIGO classification between dapagliflozin and non-dapagliflozin groups**. ** *p* < 0.05.

**Fig. 5.**
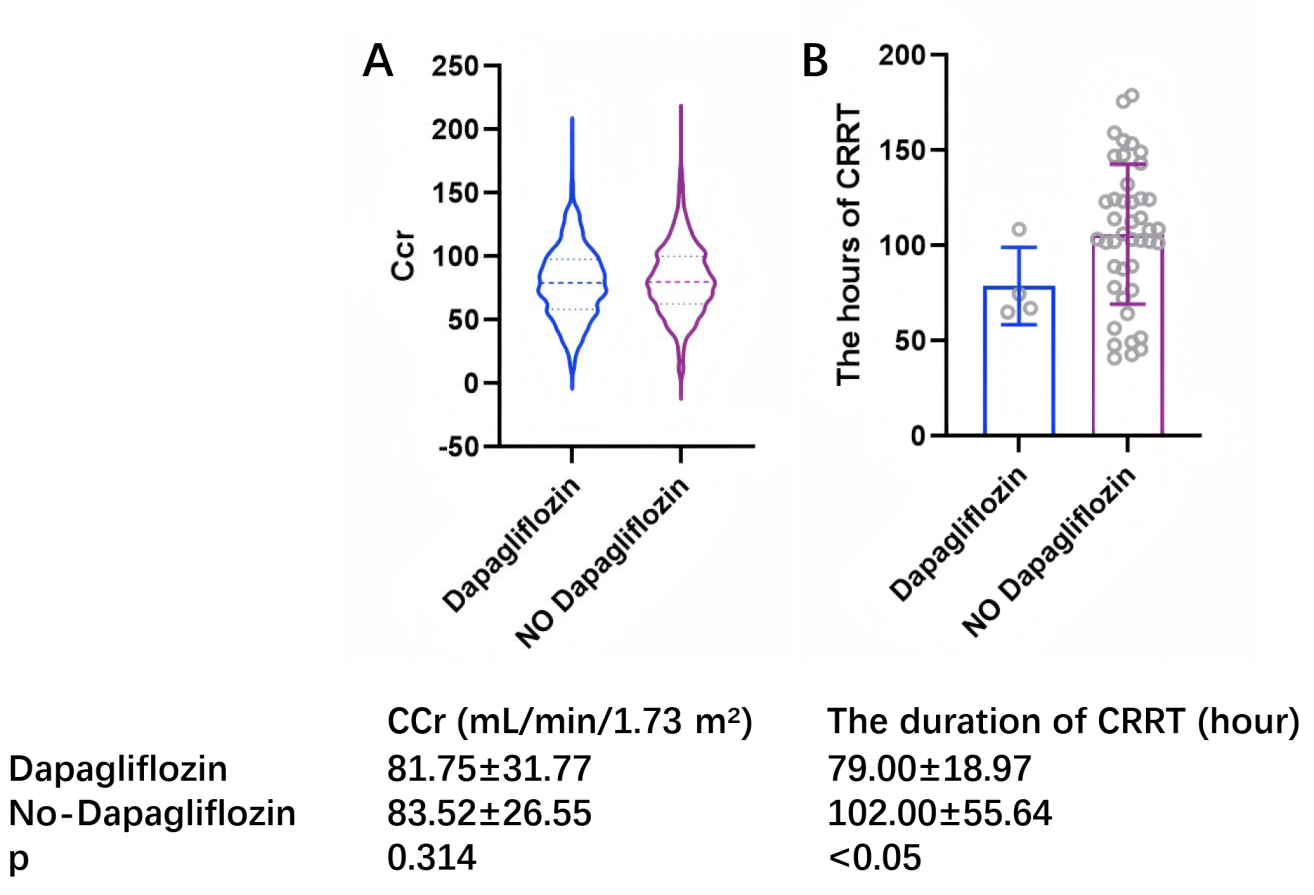
**Creatinine clearance and duration of CRRT between dapagliflozin group and non-dapagliflozin group**. (A) Represents the difference in Ccr between the Dapagliflozin and non-Dapagliflozin groups. (B) Represents the difference in the duration of CRRT between the Dapagliflozin and non-Dapagliflozin groups. CCr, creatinine clearance; CRRT, continuous renal replacement therapy.

Significant differences were observed between patients in the dapagliflozin and nondapagliflozin groups at each level of the renal impairment classification according to the Kidney Disease Improving Global Outcomes (KDIGO) criteria (Fig. 4). Preoperative treatment with dapagliflozin significantly reduced renal injury at all levels. The difference in creatinine clearance (Ccr) and the duration of CRRT between the two groups suggests a protective effect of dapagliflozin on renal function following OPCABG. The noticeable difference in the duration of CRRT for AKI patients is shown in Fig. 5, as the SGLT2i group was associated with a shorter duration of CRRT than the non-SGLT2i group after OPCABG (OR, 0.272 [95% CI, 0.097–0.762], *p* < 0.05).

## 4. Discussion

SGLT2 inhibitors have been a major breakthrough in diabetes treatment, with growing evidence demonstrating their efficacy in delaying eGFR deterioration and protecting against the progression of chronic kidney disease [14, 15, 16, 17]. However, potential associations with the risk of acute kidney injury require careful monitoring [18]. Our investigation of 15,802 coronary artery disease patients receiving off-pump CABG surgery demonstrated that SGLT2 inhibitor use enhanced postoperative renal recovery, lowered mortality rates during hospitalization, reduced the incidence of postoperative atrial fibrillation, and decreased dependency on continuous renal replacement therapy, intra-aortic balloon pumps, and extracorporeal membrane oxygenation, while also shortening overall hospital stays.

Acute kidney injury is generally categorized based on the extent of deterioration of renal function. This classification relies on measuring either the velocity of serum creatinine elevation or urinary output variations observed within 48-hour to 7-day monitoring periods [19]. AKI has now been identified as a significant contributor to increased cardiac complications post-cardiac surgery, progression to chronic kidney disease, and development of end-stage renal pathology. The clinical staging of AKI (grades I–III) substantially influences both patient outcomes and healthcare expenditures [3, 20]. Comprehensive analyses of existing research data are presented in various review articles. A major pooled data study indicates that nearly one-third of coronary artery disease patients receiving off-pump coronary artery bypass grafting developed AKI [21, 22]. The surgical mortality risk approaches 40% in individuals experiencing acute kidney injury during the perioperative period. Multiple investigations have demonstrated strong associations among preserved myocardial performance, preoperative blood glucose management, and whole-body inflammatory responses.

Multiple randomized clinical trials have extensively recorded the effects of SGLT2is on acute kidney injury [23, 24, 25]. Individuals receiving SGLT2 inhibitor therapy showed favorable effects regarding renal outcomes. These medications exhibit enhanced protective capabilities for kidney function among those with acute kidney impairment [23, 24, 25]. Two additional systematic reviews corroborated these findings, revealing that combination therapy with SGLT2 inhibitors in AKI patients having concurrent diabetes resulted in reduced AKI compared to monotherapy approaches [11, 24]. These observations align with previous research outcomes, suggesting potential renal protective benefits of SGLT2 inhibitors against postoperative AKI after off-pump coronary artery bypass grafting. Additional extensive randomized trials are necessary to confirm the therapeutic value of these agents for AKI management following off-pump coronary artery bypass procedures.

Current research predominantly centers around SGLT2 inhibitor applications for AKI management in diabetic populations, where their therapeutic application is well-documented [26], though this specific indication limits broader population generalizations [15]. Emerging findings increasingly highlight the benefits of SGLT2 inhibitors for AKI treatment in non-diabetic cardiac patients, supported by a growing body of clinical evidence [27]. This investigation identified substantial variability when assessing SGLT2 inhibitor efficacy for renal injury in non-diabetic cohorts, potentially stemming from variations in clinical protocols and differential utilization of angiotensin converting enzyme (ACE) inhibitors/angiotensin receptor blockers (ARBs) alongside SGLT2 agents, necessitating judicious clinical application [28, 29]. Comparative analysis demonstrated marginally elevated AKI risk in patients receiving renin-angiotensin system blockers versus those not prescribed these agents [30]. Pharmacological RAS suppression induces vasodilation in efferent renal arterioles, altering glomerular hemodynamics through this mechanism.

In patients undergoing off-pump coronary artery bypass grafting, preoperative administration of sodium-glucose cotransporter-2 inhibitors demonstrated a marked decrease in the occurrence of postoperative acute kidney injury. This nephroprotective effect was observed uniformly across patient subgroups with diverse comorbidities such as chronic hypertension, diabetes, and cardiac insufficiency. The investigation identified that diminished glomerular filtration pressure might potentially elevate the risk of AKI through mechanisms involving preglomerular vasoconstriction and compensatory glomerular expansion mediated by the renin-angiotensin system [31, 32]. The analysis demonstrated that when angiotensin-converting enzyme inhibitors or angiotensin receptor blockers were appropriately adjusted in preoperative regimens, SGLT2 inhibition correlated with improved renal outcomes. The clinical implications of these findings suggest potential applications for SGLT2i utilization strategies in coronary artery disease patients scheduled for cardiac revascularization procedures. However, comprehensive mechanistic studies remain necessary to elucidate the precise pathophysiology of renal impairment associated with SGLT2 inhibition during cardiovascular interventions.

## 5. Limitations of the study

This investigation examined the effects of SGLT2 inhibitors on acute kidney injury development in post-OPCABG patients. Several important constraints merit consideration in this research. The primary methodological restriction stems from the non-randomized design and possible persistence of unmeasured confounding variables, necessitating confirmation through future large-scale randomized controlled trials. Furthermore, the current analysis lacks longitudinal follow-up data to assess patient outcomes over extended periods, highlighting the need for comprehensive RCTs to evaluate chronic prognostic implications. The retrospective observational nature of this work inherently limits mechanistic exploration of therapeutic interventions. Subsequent experimental investigations employing cellular and animal models should focus on elucidating the biological pathways mediating clinical outcomes, as current hypotheses about treatment mechanisms require further substantiation. External validation through additional clinical studies remains essential to confirm the preventive efficacy of SGLT2 inhibitors against AKI in this surgical population. Due to the sample size of the studies included in the Meta-analysis, publication bias could not be completely eliminated through Egger’s test. To address this issue in the future, we will expand the research and increase the sample size to minimize publication bias as much as possible. Finally, although we performed a propensity score-matched analysis in our primary cohort to mitigate confounding, a formal risk of bias assessment for the included studies using standardized tools (e.g., ROBINS-I, Cochrane RoB 2) was precluded by insufficient reporting of methodological details in the original publications. This limitation inherently affects the strength of the conclusions that can be drawn from the meta-analytic results and underscores the need for future high-quality, prospectively designed studies with detailed reporting to confirm these findings.

## 6. Conclusion

In patients undergoing OPCABG, preoperative SGLT2i treatment was associated with a significantly reduced risk of postoperative acute kidney injury. This benefit was consistent across subgroups with varying comorbidities including hypertension, diabetes, and heart failure. Our results may be helpful in making decisions regarding the use of SGLT2is in CAD patients before OPCABG. These results need to be verified in future randomized controlled trials.

## Data Availability

The data that support the findings of this study are available on request from the corresponding author, [XBY], upon reasonable request.

## Abbreviations

SGLT2is, Sodium‒glucose cotransporter 2 inhibitors; AKI, acute kidney injury; OPCABG, off-pump artery bypass grafting; CAD, coronary artery disease; PCI-AKI, percutaneous coronary intervention-associated acute kidney injury; BMI, body mass index; eGFR, estimated glomerular filtration rate; Cr, blood creatinine; ICU, intensive care unit; CRRT, continuous renal replacement therapy; PSM, propensity score matching; PRISMA, Systematic Reviews and Meta-Analyses; IABP, intra-aortic balloon pump; Ccr, creatinine clearance; KDIGO, Kidney Disease Improving Global Outcomes; RCTs, randomized controlled trials; ACE, angiotensin-converting enzyme; ARBs, angiotensin receptor blockers.

## Supplementary Material

Supplementary material associated with this article can be found, in the online version, at https://doi.org/10.31083/RCM39400.
