# Supplementary material for: Impact of Sodium–Glucose Cotransporter 2 Inhibitors on Acute Kidney Injury Post Off-Pump Coronary Artery Bypass Grafting: A Retrospective Cohort Study and Meta-Analysis
Source: Rev Cardiovasc Med. 2026 Jan 21;27(1):39400. doi: 10.31083/RCM39400 (PMC12873702; doi:10.31083/RCM39400)
Supplement: Supplementary file 1 [file 2153-8174-27-1-39400-s1.zip › Supplementary Material.docx]

| Impact of Sodium–Glucose Cotransporter 2 Inhibitors on Acute Kidney Injury Post Off-Pump Coronary Artery Bypass Grafting: A Retrospective Cohort Study and Meta-Analysis Supplementary Fig. 1 PRISMA diagram |
| --- |
|  |
| 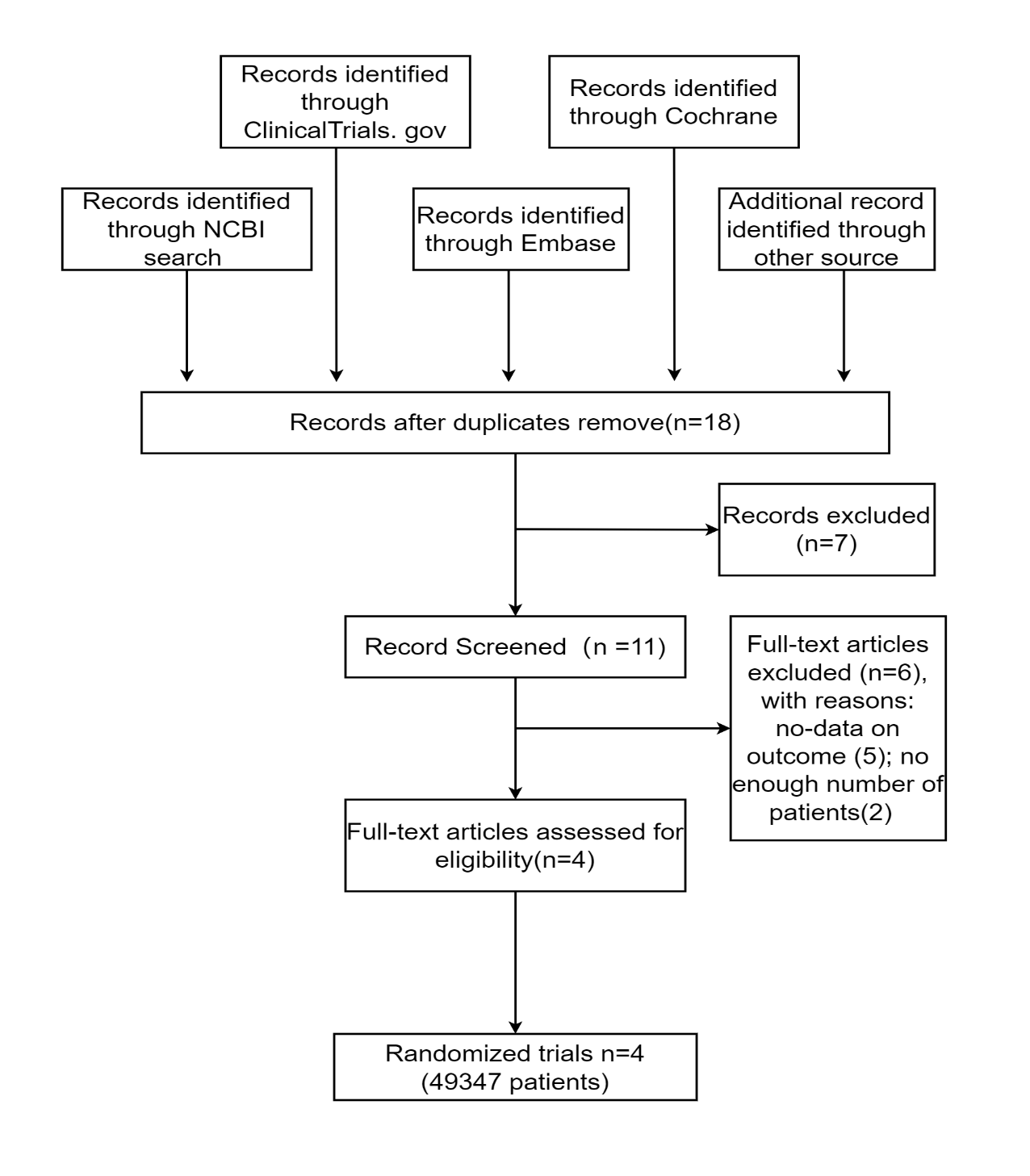 |

### Supplementary Table 1 Search strategy

| Search strategy |  |
| --- | --- |
| PubMed | (dapagliflozin) AND (acute kidney injury OR acute kidney damage OR acute kidney failure OR acute renal failure OR hypotension OR hypovolemia OR dehydration OR real world OR propensity OR observational OR randomized OR randomisation OR placebo OR Clinical Trialptyp) |
| ClinicalTrials.gov | dapagliflozin |
| EMBASE | (dapagliflozin) AND (acute kidney injury OR acute kidney damage OR acute kidney failure OR acute renal failure OR hypotension OR hypovolemia OR dehydration OR real world OR propensity OR observational OR randomized OR randomisation OR placebo OR Clinical Trial) |
| Cochrane Library | (dapagliflozin) AND (acute kidney injury OR acute kidney damage OR acute kidney failure OR acute renal failure OR hypotension OR hypovolemia OR dehydration OR real world OR propensity OR observational OR randomized OR randomisation OR placebo OR Clinical Trial) |

### Supplementary Table 2 Search strategy

| The name of Study | First author | publication year | Study design | Country | Patient population | Type of SGLT2 inhibitor |
| --- | --- | --- | --- | --- | --- | --- |
| DELIGHT | Dr. Amit X. Garg | 2023 | RCT | London Health Sciences Centre, LHSC | 293 | Dapagliflozin |
| DECLARE-TIM58 | Stephen D. Wiviott, MD；Itamar Raz, MD | 2015 | CVOT | North America (the United States and Canada), Europe (multiple countries), Latin America, the Middle East (especially Israel), Africa, and the Asia-Pacific region | 17143 | Dapagliflozin |
| DAPA-HF | John J.V. McMurray, MD；Scott D. Solomon, MD | 2017 | RCT | Europe, North America, South America and Asia | 4744 | Dapagliflozin |
| DAPA-MI(fourth) | Lars Køber, MD, DMSc  Anna Maria Langkilde, MD, PhD | 2020 | RCT | Sweden, Denmark,Norway, and the United Kingdom | 2,7167 | Dapagliflozin |

Supplementary Table 3 Clinical outcome and postoperative complication

| Variable | Dapagliflozin  (n=410) | No Dapagliflozin  (n=12442) | The all  (n=12825) |
| --- | --- | --- | --- |
| Death n(%) | 5 (1.2%) | 352 (2.8%) | 357 (2.9%) |
| AKI n(%) | 54 (13.2%) | 3651 (29.3%) | 427 (28.9%) |
| The duration in hospital (d) | 14.02±5.45 | 14.91±5.36 | 14.04±5.45 |
| POAF n(%) | 117 (28.5%) | 3824 (30.7%) | 3941 (30.7%) |
| Second operation n(%) | 8 (2%) | 185 (1.5%) | 193 (1.5%) |
| The cost of treatment (RMB) | 135087.38±315.29 | 125094.16±373.9 | 125412.96±371.76 |
| IABP n(%) | 12 (2.9%) | 492 (4%) | 504 (3.9%) |
| The duration of AKI (d) | 1.48±0.38 | 3.16±1.16 | 2.74±1.81 |
| the success of treatment of AKI n(%) | 398 (98.8%) | 1171 (96.9%) | 1569 (97.3%) |
| ECMO n(%) | 51 (0.4%) | 0 (0%) | 51 (0.4%) |

AKI indicates acute kidney injury; POAF, postoperative atrial fibrillation; CRRT, continuous renal replacement therapy; IABP, intraaortic balloon pump; ECMO, extra corporeal membrane oxygenation.
